# Determination of Carbonyl Compounds in Different Work Environments: Comparison between LC-UV/DAD and LC–MS/MS Detection Methods

**DOI:** 10.3390/ijerph191912052

**Published:** 2022-09-23

**Authors:** Federica Castellani, Arianna Antonucci, Ivano Pindinello, Carmela Protano, Matteo Vitali

**Affiliations:** 1 Department of Public Health and Infectious Diseases, Sapienza University of Rome, P.le Aldo Moro, 5, 00185 Rome, Italy; 2 Department of Ecological and Biological Sciences, Tuscia University, Largo dell’Università snc, 01100 Viterbo, Italy

**Keywords:** airborne formaldehyde, airborne carbonyl compounds, occupational exposure, analytical method, liquid chromatography

## Abstract

There were two analytical methods for the determination of 12 carbonyl compounds (CCs) by using liquid chromatography (LC) coupled with mass spectrometry (MS/MS) and diode array detector (UV/DAD) that were developed and applied to 52 samples that were collected in 10 workplaces. Linearity (0.996 < R^2^ < 0.999), intra-day repeatability (0.7 < RSD% < 10), and inter-day repeatability (5 < RSD% < 16) were acceptable for both techniques, but the highest sensibility of the MS/MS method allowed us to correctly quantify 98% of the samples (versus 32% by UV/DAD). The comparison of the concentrations that were obtained by quantifying the same sample with both techniques showed good agreement for acetaldehyde and formaldehyde (0.1 < % deviation < 30) but much higher for the less abundant congeners. In real samples, formaldehyde was the most abundant congener (concentrations between 2.7 and 77 µg m^−3^), followed by acetaldehyde (concentrations between 1.5 and 79 µg m^−3^) and butyraldehyde (concentrations between 0.4 and 13 µg m^−3^). In all the beauty salon samples, instead, the most abundant congener was acetaldehyde (concentrations between 19 and 79 µg m^−3^), probably associated with the use of beauty products. Principal components analysis (PCA) confirms the ubiquitous character of formaldehyde and highlights the influence of minority CCs on different workplaces.

## 1. Introduction

Carbonyl compounds (CCs), such as aldehydes and ketones, are ubiquitous pollutants that are among the most widespread in the environment [1,2]. Airborne CCs are primarily emitted by anthropogenic sources, such as the incomplete combustion of fossil fuels or biomass burning activities [3]. When released into the environment, CCs play an important role in the atmospheric chemistry as the precursors to several species are involved in photochemical smog (e.g., free radicals, ozone and peroxyacyl nitrates) [4].

In the last years, CCs have received more normative, scientific, and public attention both for their active role in atmospheric chemistry and for their association with several adverse effects on human health [5]. Among CCs, formaldehyde and acetaldehyde have particular health concerns; indeed, formaldehyde is a well-known sensory irritant compound, especially for sensitive individuals and it has been associated with allergies and negative outcomes for the respiratory system, while acetaldehyde can irritate the skin, eyes, and nose [6]. Besides, the International Agency for Research on Cancer (IARC) classified formaldehyde and acetaldehyde as human carcinogens (Group 1) and possibly carcinogenic to humans (Group 2B), respectively [7,8,9]. Indoor environments increase the risk of adverse health effects that are related to the exposure to formaldehyde and acetaldehyde because it is well-known that the indoor concentrations of pollutants can be two to five times higher than the respective outdoor concentrations [10,11]. In addition, the quality of indoor air is of particular importance because the general population spends the majority of the time in indoor environments, both residential or non-residential [12]. Particularly relevant is the exposure of workers that are employed in the manufacture of formaldehyde or formaldehyde-containing products, healthcare professionals, firefighters, beauticians and hairdressers, printing-rooms workers, professionals of gross anatomy and pathology laboratories, veterinarians, and embalmers [13]. Given the ubiquitous nature of formaldehyde, the World Health Organization (WHO) indicated that occupational exposure occurs in all workplaces [5], mainly through the inhalation route, but also through the dermal route [14]. Acetaldehyde is the other majority aldehyde that was revealed in the workplace, whose presence is related to the use of cosmetic products, combustion appliances, cigarette smoking, and special adhesives [15,16]. In addition to formaldehyde and acetaldehyde, other CCs that are associated with different emission sources may be present in work environments [17]. For example, crotonaldehyde is synthesized and used in the field of industry [18], propionaldehyde is emitted by housing and furniture, and butyraldehyde, hexanal, heptanal octanal, and nonanal are associated with the use of solvents, paints, perfumes, and flavoring agents [15]. Several studies focused their attention on the appearance of adverse effects on human health due to combined exposure to different CCs [19,20], that can lead both to the formation of DNA adducts and to DNA damaging [21].

Due to their impact on human health, a simple, fast, and highly effective collection sampling method is required for CCs routine determination. Generally, the analytical approaches are limited to formaldehyde and acetaldehyde determination; however, given the potential toxicity of other CCs and the synergic effects deriving from the exposure to mixture of CCs, an analytical procedure for simultaneously measuring the levels of the most common airborne CCs would be very useful. The aims of this study were: 1. to optimize an analytical procedure useful for determining the concentrations of 12 CCs; and 2. to study the distribution of 12 CCs in ten different work environments. For this purpose, the air levels of these 12 CCs were measured in ten different work environments among those that were recognized as determining potential exposure to these compounds: six wards of four different Italian hospitals, a copy shop, a beauty salon, a pharmacy, and a chemistry laboratory. The sampling was carried out by portable sampling pumps and adsorbing and derivatizing cartridges, then treated and analyzed by liquid chromatography (LC) coupled both to a spectroscopic detector (diode array detector, DAD) and tandem mass spectrometer (MS/MS) in order to optimize the two different detection methods. The performances and the results of the two detection methods were compared to define their applicability in different exposure scenarios.

## 2. Materials and Methods

### 2.1. Chemical and Materials

LC-MS grade water, acetonitrile (ACN), acetic acid, and ammonium formate were supplied by Carlo Erba (Milan, Italy), while the 12 Carbonyl-DNPH Derivatives standard solution (Formaldehyde-DNPH, Fo-DNPH; Acetaldehyde-DNPH, Ace-DNPH; Propionaldehyde-DNPH, Pro-DNPH; Crotonaldehyde-DNPH, Cro-DNPH; Butyraldehyde-DNPH, Bu-DNPH; Cyclohexanone-DNPH, Cy-DNPH; Valeraldehyde-DNPH, Val-DNPH; Hexanal-DNPH, Hex-DNPH; Heptanal-DNPH, Hep-DNPH; Octanal-DNPH, Oct-DNPH; Nonanal-DNPH, Non-DNPH; Decanal-DNPH, Dec-DNPH) by Agilent Technologies S.r.l. (Milan, Italy). Working standard solutions were prepared daily by dilution in ACN and kept in amber vials at +4 °C. Sample filtration was carried out by Choice™ PTFE Syringe Filters (0.22 μm × 13 mm, Thermo Scientific, Waltham, MA, USA). The used dual-bed sampling cartridges, coated with 2,4-dinitrophenylhydrazine (DNPH) and 1,2-bis(2-pyridyl) ethylene (BPE) for the CCs derivatization to form stable hydrazone derivatives and the removal of negative ozone interference (130 mg 2-BPE coated silica, 270 mg DNPH-coated silica), were purchased from Supelco Analytical (Bellefonte, PA, USA). A total of six SKC AirChek^®^ TOUCH sampling pumps (SKC, Eighty Four, PA, USA) were used, calibrated by a DryCal DC-lite primary flowmeter (Bios International Corporation, Butler, NJ, USA). The used analytical column was an Acclaim Carbonyl C18 RSLC (150 × 3 mm, 3 µm), acquired by Thermo Scientific (Waltham, MA, USA).

### 2.2. Instrumentation

LC analyses were carried out on an HPLC 1260 Infinity II system (Agilent Technologies Italy S.p.A. Milan, Italy) that was equipped with high pressure mixing and an autosampler Agilent G7129A. The MS/MS determinations were performed by using a QTRAP 5500 mass spectrometer with an ESI source, operating in MRM (multiple reaction monitoring) mode (AB SCIEX S.r.l. Forster City, CA, USA). Analyses were achieved in negative mode. The DAD determinations were performed by UV-DAD 1290 DAD FS (Agilent Technologies Italy S.p.A. Milan, Italy), set at 360 nm. The used measuring cell had an optical path of 1 mm and a volume of 1 µL.

### 2.3. Sampling Sites and Strategy

The indoor carbonyl levels were measured in ten different workplaces, selected based on a recent systematic review [13]. The general information and characteristics of each sampling are reported in Table 1. Briefly, for each working environment, four to six sample pumps were used both as personal samplers, in order to control employees’ exposure, and as environmental samplers, in order to estimate indoor contamination. In every sampling site, the assessment of CCs’ background levels was made by placing a sampling pump outside and near the monitoring site. Air samples were collected into the dual-bed cartridges at a flow rate of 0.14 L min^−1^. Sampling was carried out during normal working hours, with sampling time ranging from 51 to 406 min (Table 1). The chosen flow rate and sampling times ensure that the collected CCs would not consume more than 30% of the DNPH that was coated on the cartridges; this was checked for every sample by LC-UV/DAD analysis at 360 nm and subsequent quantification of unreacted DNPH (Supplementary Material S1). A preliminary test was performed in order to assess that the chosen sampling flow rate did not consume more than 30% of DNPH. Analysis of the blank samples (i.e., 5 unsampled cartridges) was performed to verify possible contaminations during extraction procedure. The portable pumps’ flow rates were checked before and after every sampling campaign using a calibrated flowmeter. The sampled DNPH-coated cartridges were stored in a refrigerator at +4 °C until the analysis, carried out within two weeks from the sampling campaign.

### 2.4. Sample Analysis

The 12 analyzed carbonyl compounds (Fo-DNPH, Ace-DNPH, Pro-DNPH, Cro-DNPH, Bu-DNPH, Cy-DNPH, Val-DNPH, Hex-DNPH, Hep-DNPH, Oct-DNPH, Non-DNPH, Dec-DNPH) were eluted from the DNPH-coated cartridge with 4.0 mL of ACN, with an elution rate of 1 mL min^−1^ as suggested by the manufacturer of the cartridges. The eluate was filtered and then injected in the LC system and detected by using both spectrometric and spectroscopic detectors.

### 2.5. Optimization of the Analytical Determinations

Both for the LC-MS/MS and LC-UV/DAD determinations, the calibration curves were constructed by using an external standard method, in the range 1.56–100 µg L^−1^ for MS/MS and 8–1000 µg L^−1^ for UV/DAD. For the quantification of real samples, the external standard quantification method was used after verifying that there was no matrix effect for both the determination techniques. Limits of detection (LODs) and quantification (LOQs) were calculated as the minimum concentration of the analyte that produces a signal-to-noise ratio greater than 3 and 10, respectively. Intra-day reproducibility, evaluated as the relative standard deviation (RSD%), was calculated on 10 repeated injections in the same day at three concentration levels:

(1) Lowest Level (L_L_): Concentration level representing an agreement between the LOQs of the detected analytes;
(2) Medium Level (M_L_): Concentration level corresponding to the highest LOQ of the detected analytes;
(3) Highest Level (H_L_): Concentration corresponding to 10 times the L_L_.

Inter-day reproducibility, instead, was obtained by performing triplicate analysis of the same standard solution for seven consecutive days. The result was expressed as RSD%.

### 2.6. Statistical Elaboration

The statistical software R (R-project for statistical computing, Ver. 3.0, 32-bit) was used to perform principal component analysis (PCA) on the 12 individual CCs’ congeners in order to illustrate the distribution pattern and the possible emission sources. Before the statistical analysis, the matrix of the data was transformed by column mean centering and row and column autoscaling in order to correct for different variable scaling and units.

## 3. Results and Discussion

### 3.1. Optimization of the Chromatographic Separation

To find the best chromatographic condition for CCs separation, we used a C18 Acclaim Carbonyl column, testing different elution gradients and flow rates. The best solution was a mobile phase consisting of (A) ammonium acetate 2 mM and (B) ACN at a flow rate of 0.8 mL min^−1^. The gradient elution started from A:B 53:47 (*v*/*v* %) for 5 min and linearly increasing to 90% B in −5 min, then isocratic elution for 5 min. The initial elution conditions were then restored for 5 min. The total duration of the analysis was 20 min. The injection volume was 5 µL. The same optimized chromatographic separation was coupled both with MS/MS and UV/DAD detectors. The retention times of the selected analytes are reported in Table 2.

### 3.2. Optimization of the Spectrometric Determination and Data Quality

To choose the precursor and product ions that are useful to quantitative and qualitative determinations and to find the best parameters for ionic transmission in mass spectrometry, the infusion of standard solution of Ccs (10 µg L^−1^ in ACN) at a flow rate of 7 µL min^−1^ were carried out in negative mode. Nebulizer, turbo, and curtain gases were respectively set at 30, 55, and 60 psi; the temperature was set at 500°C; and declustering and entrance potential were set at −60 and −10, respectively. The optimized parameters for each analyte are reported in Table 2, together with precursor and product ions.

The validation parameters of the LC-MS/MS optimized method are shown in Table 3. The method linearity in the investigated concentration range (1.56–100 µg L^−1^), expressed as R^2^, was between 0.999 and 0.996 for all the studied analytes. The values of intra-day repeatability (RSD%) calculated at the lowest level (L_L_, 0.20 µg L^−1^) ranged from 3.0% to 7.8%. The RSD% were found to be lower at higher concentrations (M_L_, 0.78 µg L^−1^ and H_L_, 20 µg L^−1^) with values between 1.5% and 3.3% for M_L_ and between 1.2% and 2.7% for H_L_. The obtained results were much lower than those that were obtained by Chi et al. [22]. The intra-day repeatability values (RSD%) were between 8.6% and 15% for L_L_, between 6.3% and 14% for M_L_, and between 7.7% and 16% for H_L_. LODs and LOQs were comparable or lower than those that were found by Chi et al. [22] and acceptable for the analytical determinations. The analytical data that were considered for the optimization of the spectrometric determination have confirmed this method to be sensitive, precise, and accurate for the evaluation of CCs in indoor air. Supplementary Material S2 shows the LC-MS/MS chromatogram that was obtained by injecting 5 µL of aldehydes-DNPH standard solution at 0.2 µg L^−1^.

### 3.3. Optimization of the Spectroscopic Determination and Data Quality

The method linearity in the investigated concentration range (8–1000 µg L^−1^), expressed as R^2^, was 0.999 for all the analytes that were under study, as reported in Table 4. Although the range of the investigated concentrations was rather wide, the results were comparable with those that were obtained by Feng et al. [23]. The values of intra-day repeatability (RSD%), calculated at lowest level (L_L_, 25 µg L^−1^), were between 2.3% and 7.8%. The RSD% were found to be lower at higher concentrations (M_L_, 62 µg L^−1^ and H_L_, 250 µg L^−1^) with values between 1.5% and 3.3% for M_L_ and between 1.2% and 2.7% for H_L_ (Table 4). The intra-day repeatability values (RSD%) were between 8.6% and 15% for L_L_, between 6.3% and 14% for M_L_, and between 7.7% and 16% for H_L_ (Table 4). The values that were obtained both for intra- and inter-day repeatability were comparable with those that were obtained for MS/MS determination, despite the different concentrations of the three examined levels. As expected, LODs and LOQs were higher than those that were obtained by MS/MS analysis but comparable or lower than those that were obtained in other works [24,25]. Supplementary Material S3 shows the LC-UV/DAD chromatogram that was obtained by injecting 5 µL of aldehydes-DNPH standard solution at 25 µg L^−1^.

### 3.4. Comparison between LC-MS/MS and LC-UV/DAD

The MS/MS detection method was compared with the UV/DAD detection at 360 nm method. All the real samples were analyzed by using both detection methods. Supplementary Material S4–S6 show the LC-MS/MS and LC-UV/DAD chromatograms that were obtained by injecting 5 µL of extracted real samples (PL5-doc, BS1-hair, and ParL4-lab). The results of three carbonyls (Fo-DNPH, Ace-DNPH, and Pro-DNPH) are listed in Table 5. Unfortunately, the comparison was possible only for three congeners because, due to the lower sensibility of the UV/DAD detection method, only lower molecular weight analytes were detected in more than 50% of the samples that were analyzed by using UV/DAD detector. Table 5 shows that for formaldehyde and acetaldehyde existed good agreement between the two detection methods with percentage deviations between 0.2 and 29% and 0.1 and 30%, respectively. For pro-DNPH, since the determined concentrations are much lower than those that were obtained for fo-DNPH and ace-DNPH, with values between 0.32 and 5 µg m^−3^, small differences can cause a considerable deviation (percentage deviations between 1.2% and 50%). The obtained percentage deviations were in line with those that were found by Chi et al. [22]. The deviations of cy-DNPH and cro-DNPH were in the range of 443–2004% and 16576–1601%, respectively; the obtained results were not acceptable, and this trend could be explained by considering the lack of selectivity of the spectroscopic method. In real samples, in fact, there could be an interference that co-elutes with the target compounds causing an increase in the signal with a consequent overestimation of the concentrations when samples were analyzed by using UV/DAD detector. This problem was overcome with the use of a high selectivity spectrometer detector operating in MRM mode. Dure to the lower LODs (Table 3 and Table 4), the quantification of low concentration’s CCs in air samples (mainly medium and high molecular weight carbonyls) was much more accurate by using the MS/MS detector than by using UV/DAD. For this reason, the real results will be described by using the concentrations that were obtained by MS/MS analysis.

### 3.5. Real Samples

Table 6 reports the concentration of the 12 CCs that were assessed in ten different work environments. The average blank concentrations (Supplementary Material S7) were subtracted from sample concentrations. Analyzing the samples with LC-MS/MS technique, only 2.4% of the samples were <LOQ (Table 6); this percentage rises to 68% when the samples were analyzed by using LC-UV/DAD. As we can see from Table 6, the highest concentration of hexanal (13 μg m^−3^) was recorded in one of the samples that was collected in the beauty salon (BS5-bea) and associated with a personal sampler of a beautician. Unfortunately, a comparison with the literature is not possible, due to the lack of data. The highest concentrations of cyclohexanone, crotonaldehyde, heptanal, and octanal (2.7 μg m^−3^, 1.8 μg m^−3^, 3. μg m^−3^, and 3.2 μg m^−3^, respectively) were recorded in the sample HL1-lab, collected in the histology laboratory (environmental sampler). In the same workplace, was also recorded the highest concentration of decanal (4.0 μg m^−3^) in a personal sampler (HL5-tech). The concentrations that were obtained in this study were comparable or much higher than those that were obtained by Lu et al. [26,27] in special rooms and different wards of Chinese hospitals. The highest concentrations of butyraldehyde, nonanal, propionaldehyde, and valeraldehyde (13 μg m^−3^, 9.9 μg m^−3^, 5.0 μg m^−3^, and 8.0 μg m^−3^, respectively) were recorded in the enclosure environment (HRL5-lab). Unfortunately, a comparison with the literature is not possible because, to our knowledge, no study was conducted in a similar work environment.

**Figure 1:**
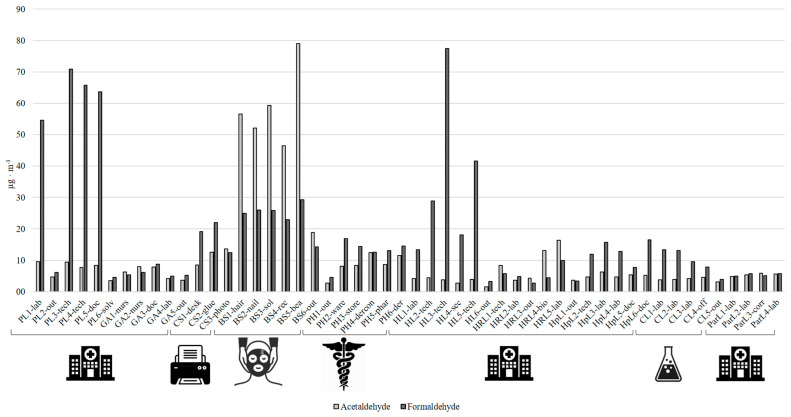
Distribution (concentrations expressed as µg m^−3^) of formaldehyde and acetaldehyde in the previously described work environments.

Formaldehyde and acetaldehyde concentrations in work environments’ indoor air ranged from 2.7 to 77 µg m^−3^ and from 1.5 to 79 µg m^−3^, respectively (Table 6). For all the sampling sites, the indoor concentrations of the two pollutants were higher than the corresponding outdoor values (Table 6). For all the sampling sites, the concentrations of formaldehyde were comparable or higher than the respective concentrations of acetaldehyde, except for the beauty salon. In all the five beauty salon’s samples, in fact, the concentrations of acetaldehyde were much higher than the respective concentrations of formaldehyde (Figure 1). This trend was also found in the beauty salon of Tehran by Hadei et al. [10], and could be due to the composition of cosmetic products that are used in the beauty salon. Evtyugina et al. [28] measured CCs’ concentrations in a beauty salon in Spain, finding formaldehyde and acetaldehyde mean concentrations of 12 and 9.0 μg m^−3^, respectively. These values are much lower than those that were determined in this work (mean concentration of 24 and 52 μg m^−3^ for formaldehyde and acetaldehyde, respectively).

**Figure 2:**
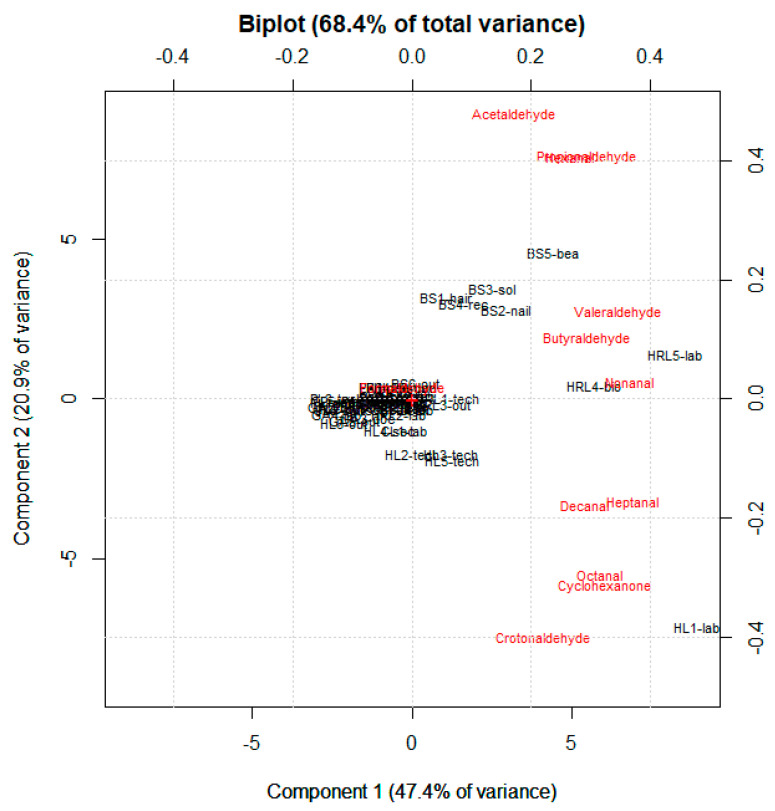
Biplot of the PCA (PC1 and PC2) that was performed on the concentration data that were yielded at each work place.

The highest concentrations of formaldehyde were detected in the hospital environments (sampling sites HL3, PL3, PL4, PL5, PL1, HL5, and HL2) where formalin solutions were used. Lower concentrations were detected in the hospital environments of gastroenterology and parasitology; in the first ward formaldehyde was not deliberately used and in the second ward a closed system was used to contain formaldehyde emissions. The maximum and minimum formaldehyde contamination levels that were detected in different hospital wards in this study were compared with the literature. The comparison showed that formaldehyde contamination levels in this work were much lower than those that were found in other Italian and international hospitals [29,30,31,32,33]. Concerning the acetaldehyde contamination levels, the values that were found in this work were comparable or much lower than the values that were found by Sousa et al. [6] and Lu et al. [26] in Brazilian and Chinese hospitals, respectively. For formaldehyde and acetaldehyde contamination levels in the copy shop, the concentrations that were found in this study were in line or lower than those that were found by Saraga et al. [34] in a printery in the center of Athens but higher than those that were found by Ho et al. [1] in a photocopy center at a Chinese university. Formaldehyde and acetaldehyde contamination levels in the selected pharmacy were in line with those that were found by Loh et al. [35] in eight different drug stores in the USA. Unfortunately, to our knowledge, there are no studies that were carried out in chemistry laboratories. However, the contamination levels that were found in this selected work environment were comparable with those that were found in the pharmacy and in some wards of the investigated hospitals (HpL and CA). Unfortunately, for the concentrations of the other CCs, a comparison with the literature is not possible given the lack of data. However, to further investigate the distribution patterns of target analytes in different workplaces, PCA has been conducted on the 12 individual CCs’ congeners and 52 samples. The PCA results are summarized in the biplot that is shown in Figure 2, while scores and loadings are shown in Table 7 and Table 8, respectively.

Two significant components (PC1 and PC2), accounting for 68.4% of the total variance, were obtained. The biplot in Figure 2 well separates four clusters of work environments, each characterized by the presence of different CCs. The first cluster that was identified on the central part of the biplot, was predominated by formaldehyde, which did not show high variability across the 44 samples. This is in agreement with the fact that formaldehyde is considered a ubiquitous indoor pollutant [36,37]. The second cluster, in the upper right of the biplot, is characterized by the presence of all the indoor beauty salon samples and three carbonyl compounds (acetaldehyde, propionaldehyde, and hexanal) that are probably associated with the use of beauty products. The third cluster to the right of the biplot is characterized by the presence of the two samples (one personal and the other environmental) that are related to the enclosure. This environment was characterized by the presence of valeraldehyde, nonanal, and butyraldehyde, maybe associated with the presence of living animals. The fourth cluster, at the bottom right of the biplot, is characterized by the presence of one environmental sample that was collected from a histology laboratory. This work environment is characterized by the presence of five carbonyl compounds (decanal, heptanal, octanal, cyclohexanone, and crotonaldehyde) that are probably associated with the use of chemicals for different purposes, such as cleaning and disinfection.

## 4. Conclusions

This study demonstrated that LC-MS/MS detection is a suitable method for the determination of carbonyl compounds in different work environments. Good selectivity, sensibility, linearity, and reproducibility were all obtained. The proposed method, compared with LC–UV/DAD detection, resulted to be the most suitable strategy for the analysis of CCs in work environments given the high sensibility and the lowest LODs. The application of the optimized method to real samples also achieved excellent results: only 2.4% of the samples resulted to be lower than LOQs. When the same samples were analyzed by using LC-UV/DAD, this percentage rises to 68%. Among most of the work environments, formaldehyde was the most abundant detected carbonyl, followed by acetaldehyde and butyraldehyde. On the contrary, the most abundant congener that was detected in the beauty salon was acetaldehyde; this trend could be attributed to the composition of cosmetic products that are used in beauty salon. The PCA that was performed on the dataset confirms the ubiquitous character of formaldehyde and highlights the different influence of minority CCs on different work environments.

## Figures and Tables

**Table 1:** Specifications of the sampling sites and conditions.

	Sampling Date	Sampling SITE	Sampling Type	Main Task/Activity	Sampling Time (min)	Volume (m^3^)
PL1-lab	28 January 2021	Pathology Laboratory	Laboratory Environment	Preparation of anatomical specimens in formalin	264	0.037
PL2-out			Environmental—External window ledge	Urban environment	266	0.037
PL3-tech			Staff—Laboratory Technician	Preparation of anatomical specimens in formalin	261	0.037
PL4-tech			Staff—Laboratory Technician	Preparation of anatomical specimens in formalin	262	0.037
PL5-doc			Staff—Ward doctor	Preparation of anatomical specimens in formalin	202	0.028
PL6-solv			Environmental—Solvent storage	Storage of formalin tanks	273	0.038
GA1-nurs	2 February 2021	Gastroenteroloy Ambulatory	Staff—Nurse	Clinical examinations—gastroscopy and colonoscopy	149	0.021
GA2-nurs			Staff—Nurse	Clinical examinations—gastroscopy and colonoscopy	146	0.020
GA3-doc			Staff—Ward doctor	Clinical examinations—gastroscopy and colonoscopy	125	0.018
GA4-lab			Laboratory Environment	Clinical examinations—gastroscopy and colonoscopy	159	0.022
GA5-out			Environmental—External window ledge	Urban environment	140	0.020
CS1-desk	30 April 2021	Copy Shop	Staff—desk	Cashier	300	0.042
CS2-glue			Staff—Gluing machine	Gluing of backs of books	300	0.042
CS3-photo			Staff—Photocopier	Use of laser photocopier	300	0.042
BS1-hair	31 April 2021	Beauty Salon	Staff—Hairdresser	Hairstyling, hair dye	351	0.049
BS2-nail			Staff—Nail Technician	Gel nails extension	352	0.049
BS3-sol			Environmental—Solarium	Tanning bed	347	0.049
BS4-rec			Staff—Receptionist	Secretarial work	343	0.048
BS5-bea			Staff—Beautician	Waxing, manicure, pedicure	334	0.047
BS6-out			Environmental—External window ledge	Urban environment	335	0.047
PH1-out	3 May 2021	Pharmacy	Environmental—External window ledge	Urban environment	375	0.053
PH2-ware			Environmental—Warehouse	Storage of pharmaceutical products	315	0.044
PH3-store			Staff—Storeman	Storage of pharmaceutical products	313	0.044
PH4-dercon			Staff—Dermocosmetic consultant	Employed to the sale	314	0.044
PH5-phar			Staff—Pharmacist	Employed to the sale	321	0.045
PH6-der			Environmental—Dermocosmetic	Presence of wooden shelves	314	0.044
HL1-lab	22 September2021	Histology Laboratory	Laboratory Environment	Preparation of anatomical specimens in formalin	406	0.057
HL2-tech			Staff—Laboratory Technician	Preparation of anatomical specimens in formalin	406	0.057
HL3-tech			Staff—Laboratory Technician	Preparation of anatomical specimens in formalin	397	0.056
HL4-sec			Staff—Secretary	Acceptance of histological samples	394	0.055
HL5-tech			Staff—Laboratory Technician	Preparation of anatomical specimens in formalin	391	0.055
HL6-out			Environmental—External window ledge	Urban environment	393	0.055
HRL1-tech	5 April 2022	Histology Research Laboratory	Staff—Laboratory and Enclosure Technician	Preparation of animal anatomical specimens in formalin and management of the enclosure	154	0.022
HRL2-lab			Laboratory Environment	Preparation of animal anatomical specimens in formalin	155	0.022
HRL3-out			Environmental—External window ledge	Urban environment	155	0.022
HRL4-bio			Staff—Biologist Head of the Enclosure	Histological sampling of animal parts and management of the enclosure	81	0.011
HRL5-lab			Laboratory environment—Enclosure	Histological sampling of animal parts	51	0.007
HpL1-out	6 April 2022	Histopathology Laboratory	Environmental—External window ledge	Urban environment	244	0.034
HpL2-tech			Staff—Laboratory Technician	Preparation of anatomical specimens in formalin	311	0.044
HpL3-lab			Laboratory Environment	Preparation of anatomical specimens in formalin	306	0.043
HpL4-lab			Laboratory Environment	Storage of anatomical specimens in formalin	302	0.042
HpL5-doc			Staff—Ward doctor	Preparation of anatomical specimens in formalin	163	0.023
HpL6-doc			Staff—Ward doctor	Preparation of anatomical specimens in formalin	251	0.035
CL1-lab	12 April 2022	Chemical Laboratory	Laboratory Environment	Environmental Samples Preparation and Storage	273	0.038
CL2-lab			Laboratory Environment	Environmental Samples Preparation and Analysis	270	0.038
CL3-lab			Laboratory Environment	Quantitative analytical chemistry	271	0.038
CL4-off			Office	Data elaboration	271	0.038
CL5-out			Environmental—External window ledge	Urban environment	270	0.038
ParL1-lab	13 April 2022	Parasitology Laboratory	Laboratory environment	Sample preparation and analysis	222	0.031
ParL2-lab			Laboratory environment	Sample preparation and analysis	221	0.031
ParL3-corr			Corridor Outside the Parasitology Laboratory	-	222	0.031
ParL4-lab			Laboratory Environment	Sample preparation and analysis	220	0.031

**Table 2:** Optimized spectrometric conditions for each studied analyte. The letter “Q” indicates the ion that was chosen as quantifier, the letter “q” indicates the ion that was chosen as qualifier.

	Retention Times (min)	Molecular Mass	Precursor Ion		Product Ion	Collision Energy (eV)	Collision Cell Exit Potential (V)
Fo-DNPH	5.83	210	209	Q	151	−11	−15
				q	163	−11	−15
Ace-DNPH	8.05	224	223	Q	151	−12	−6
				q	163	−12	−10
Pro-DNPH	9.68	238	237	Q	163	−15	−15
				q	151	−15	−15
Cro-DNPH	10.36	250	249	Q	172	−16	−15
				q	151	−16	−15
Bu-DNPH	10.65	252	251	Q	163	−15	−15
				q	151	−15	−15
Cy-DNPH	11.29	278	277	Q	247	−15	−15
				q	231	−24	−15
Val-DNPH	11.40	266	265	Q	163	−15	−15
				q	152	−25	−15
Hex-DNPH	12.06	280	279	Q	152	−25	−15
				q	163	−15	−15
Hep-DNPH	12.73	294	293	Q	163	−15	−15
				q	152	−25	−15
Oct-DNPH	13.53	308	307	Q	163	−19	−15
				q	205	−19	−15
Non-DNPH	14.53	322	321	Q	163	−20	−15
				q	205	−20	−15
Dec-DNPH	15.85	336	335	Q	163	−19	−15
				q	171	−19	−15

**Table 3:** LODs (µg L^−1^), LOQs (µg L^−1^), linearity (R^2^), and the intra- and inter-day repeatability (RSD%) that were obtained from the LC-MS/MS optimized method. L_L_, M_L_, and H_L_ correspond respectively to 0.20, 0.78, and 20 µg L^−1^.

			Linearity	Intra-Day Repeatability (n = 10)			Inter-Day Repeatability (n = 7)		
	LOD	LOQ	R^2^	L_L_	M_L_	H_L_	L_L_	M_L_	H_L_
Fo-DNPH	0.39	0.78	0.998	7.8	3.0	1.3	14	6.5	6.5
Ace-DNPH	0.39	0.78	0.999	6.8	2.9	1.2	10	6.3	7.7
Pro-DNPH	0.049	0.20	0.998	3.0	2.2	1.4	11	6.6	8.5
Cro-DNPH	0.049	0.20	0.998	3.6	3.3	1.4	8.8	7.3	9.4
Bu-DNPH	0.098	0.39	0.998	5.8	2.1	1.5	11	7.1	9.3
Cy-DNPH	0.012	0.024	0.997	3.5	2.5	2.3	15	12	13
Val-DNPH	0.024	0.098	0.998	4.6	2.7	2.6	11	9.2	11
Hex-DNPH	0.012	0.049	0.997	3.5	2.3	2.2	11	10	13
Hep-DNPH	0.012	0.098	0.997	2.3	1.9	1.8	13	14	16
Oct-DNPH	0.098	0.39	0.997	3.4	2.7	2.3	12	13	15
Non-DNPH	0.024	0.098	0.996	5.4	3.3	2.7	12	14	15
Dec-DNPH	0.049	0.20	0.997	3.7	1.5	2.6	8.6	9.8	11

**Table 4:** LODs (µg L^−1^), LOQs (µg L^−1^), linearity (R^2^), and the intra- and inter-day repeatability (RSD%) that were obtained from the LC-UV/DAD optimized method. L_L_, M_L_, and H_L_ correspond respectively to 25, 62, and 250 µg L^−1^.

			Linearity	Intra-Day Repeatability (n = 10)			Inter-Day Repeatability (n = 7)		
	LOD	LOQ	R^2^	L_L_	M_L_	H_L_	L_L_	M_L_	H_L_
Fo-DNPH	16	62	0.999	10	4.9	1.6	9.3	11	9.5
Ace-DNPH	12	62	0.999	6.0	4.0	1.8	6.5	8.3	8.8
Pro-DNPH	8	31	0.999	5.8	2.7	1.0	5.6	7.6	9.3
Cro-DNPH	8	31	0.999	7.8	3.2	1.7	6.1	7.8	8.8
Bu-DNPH	8	31	0.999	3.8	1.3	0.80	5.6	7.2	9.2
Cy-DNPH	8	25	0.999	2.3	2.0	0.85	5.2	7.6	9.4
Val-DNPH	8	25	0.999	4.4	1.9	0.74	5.4	7.2	9.1
Hex-DNPH	8	25	0.999	5.6	1.8	1.3	5.0	6.8	9.2
Hep-DNPH	8	31	0.999	7.0	3.2	1.3	7.1	6.8	8.1
Oct-DNPH	8	31	0.999	6.1	3.7	1.2	6.3	6.5	9.3
Non-DNPH	12	62	0.999	8.2	5.6	1.0	10	7.1	9.3
Dec-DNPH	16	62	0.999	9.5	2.5	1.5	11	6.7	9.0

**Table 5:** Percentage deviations of real samples that were obtained from the comparison of ESI-MS/MS and UV/DAD detection methods.

	Formaldehyde	Acetaldehyde	Propionaldehyde
PL1-lab	18	18	-
PL2-out	21	20	1.2
PL3-tech	3.9	0.5	-
PL4-tech	11	4.7	
PL5-doc	2.3	1.4	-
PL6-solv	0.5	0.6	-
GA1-nurs	12	16	-
GA2-nurs	18	24	-
GA3-doc	15	19	-
GA4-lab	13	14	-
GA5-out	7.0	22	-
CS1-desk	2.1	19	24
CS2-glue	3.8	16	14
CS3-photo	16	24	28
BS1-hair	14	13	31
BS2-nail	2.2	25	35
BS3-sol	12	26	31
BS4-rec	16	30	31
BS5-bea	29	30	31
BS6-out	21	11	23
PH1-out	11	6.0	35
PH2-ware	7.8	10	40
PH3-store	19	19	42
PH4-dercon	18	21	48
PH5-phar	23	22	44
PH6-der	12	11	47
HL1-lab	25	13	29
HL2-tech	3.4	9.7	5.3
HL3-tech	1.0	5.0	8.5
HL4-sec	4.2	16	22
HL5-tech	0.7	4.2	5.0
HL6-out	5.6	25	-
HRL1-tech	13	2.3	-
HRL2-lab	20	6.4	-
HRL3-out	18	30	-
HRL4-bio	7.1	18	-
HRL5-lab	7.1	1.6	-
HpL1-out	25	20	-
HpL2-tech	12	0.1	39
HpL3-lab	5.2	0.7	9.2
HpL4-lab	2.0	4.0	-
HpL5-doc	0.3	15	-
HpL6-doc	3.8	9.1	-
CL1-lab	1.2	0.1	-
CL2-lab	7.3	7.7	-
CL3-lab	0.2	3.1	49
CL4-off	8.1	3.7	50
CL5-out	12	6.3	47
ParL1-lab	26	0.4	-
ParL2-lab	6.1	0.3	-
ParL3-corr	7.3	15	-
ParL4-lab	8.3	3.2	-

**Table 6:** Concentration of the 12 Ccs (µg m^−3^) that were determined in the 10 different work environments.

	Acetaldehyde	Butyraldehyde	Cyclohexanone	Crotonaldehyde	Decanal	Heptanal	Hexanal	Formaldehyde	Nonanal	Octanal	Propionaldehyde	Valeraldehyde
PL1-lab	9.5	0.46	0.01	<LOQ	0.08	0.03	0.19	55	0.49	0.08	0.67	0.17
PL2-out	4.7	0.57	0.08	<LOQ	0.12	0.06	0.06	6.1	0.73	0.15	0.95	0.23
PL3-tech	9.4	0.89	0.01	<LOQ	0.15	0.03	0.21	71	0.55	0.09	0.92	0.21
PL4-tech	7.7	1.4	0.11	<LOQ	0.11	0.06	0.19	66	0.62	0.12	0.99	0.71
PL5-doc	8.4	0.80	0.02	<LOQ	0.18	0.02	0.18	64	0.63	0.11	0.89	0.22
PL6-solv	3.5	0.76	0.05	<LOQ	0.17	0.06	0.08	4.5	0.82	0.14	0.87	0.29
GA1-nurs	6.2	0.81	0.11	<LOQ	0.19	0.04	<LOQ	5.3	0.81	0.13	0.74	0.25
GA2-nurs	7.9	0.61	0.12	<LOQ	0.14	0.00	0.02	6.1	0.63	0.09	0.84	<LOQ
GA3-doc	7.9	0.68	0.26	0.02	0.70	0.11	0.19	8.8	1.7	0.43	1.1	0.03
GA4-lab	4.2	0.41	0.13	<LOQ	0.25	0.01	<LOQ	5.0	0.68	0.08	0.63	0.04
GA5-out	3.7	0.38	0.16	0.03	0.46	0.14	0.14	5.2	1.9	0.23	0.72	0.04
CS1-desk	8.5	1.2	0.06	0.08	1.0	0.52	2.2	19	2.2	0.56	1.2	0.95
CS2-glue	13	0.82	0.15	0.06	1.1	0.50	2.2	22	2.2	0.57	1.2	0.71
CS3-photo	14	0.98	0.07	0.05	0.72	0.33	1.4	12	1.7	0.38	1.1	0.58
BS1-hair	57	1.9	0.11	0.01	1.0	0.51	6.5	25	2.4	0.01	4.0	0.64
BS2-nail	52	1.7	0.12	0.01	1.7	0.88	7.8	26	3.5	0.91	4.4	2.3
BS3-sol	59	1.8	0.14	0.01	1.1	0.75	8.5	26	3.2	0.58	4.3	2.4
BS4-rec	46	1.8	0.07	0.01	0.79	0.57	7.4	23	2.2	0.44	3.9	2.6
BS5-bea	79	2.4	0.07	0.03	1.6	1.1	13	29	4.5	1.0	4.7	2.9
BS6-out	19	1.7	0.05	0.01	0.62	0.67	2.8	14	2.5	0.58	1.7	1.5
PH1-out	2.7	0.44	0.12	0.02	0.39	0.12	0.32	4.5	1.1	0.20	1.8	0.19
PH2-ware	8.1	1.1	0.27	0.02	0.70	0.34	2.0	17	2.5	0.39	1.6	0.87
PH3-store	8.4	1.7	0.15	0.02	0.73	0.31	2.2	14	1.9	0.40	1.8	1.3
PH4-dercon	12	1.2	0.09	0.02	0.65	0.42	2.7	13	2.5	0.42	1.9	0.97
PH5-phar	8.7	1.6	0.18	0.02	0.93	0.33	2.2	13	2.0	0.45	1.6	1.5
PH6-der	11	1.4	0.10	0.02	0.49	0.24	1.8	15	1.5	0.29	1.8	0.96
HL1-lab	4.2	2.8	2.7	1.8	3.4	3.0	3.1	13	4.7	3.2	2.1	2.6
HL2-tech	4.4	0.76	0.18	0.01	1.1	0.42	0.80	29	2.4	2.2	0.60	0.37
HL3-tech	3.7	0.73	0.14	0.004	2.3	0.82	1.2	77	5.4	1.8	0.64	0.51
HL4-sec	2.7	0.93	0.14	0.004	1.3	0.36	0.98	18	2.5	0.74	0.61	0.53
HL5-tech	3.9	1.17	0.22	0.005	4.0	0.36	1.0	42	3.9	1.6	0.69	0.72
HL6-out	1.5	0.45	0.04	0.002	0.50	0.15	0.32	3.3	1.0	0.25	0.32	0.15
HRL1-tech	8.4	3.8	0.52	0.02	0.75	0.50	1.80	5.7	3.9	0.64	2.0	2.4
HRL2-lab	3.6	1.7	0.19	0.01	0.68	0.22	0.20	4.9	4.0	0.63	1.5	0.54
HRL3-out	4.3	2.5	0.55	0.02	0.60	0.63	2.5	2.7	3.3	0.63	1.8	2.9
HRL4-bio	13	8.1	1.3	0.02	1.4	1.2	4.2	4.4	6.5	1.1	3.1	7.0
HRL5-lab	16	13	1.30	0.04	1.6	1.2	4.6	10	9.9	1.6	5.0	8.0
HpL1-out	3.6	2.6	0.29	0.01	0.30	0.20	0.61	3.3	1.5	0.30	1.8	1.3
HpL2-tech	4.7	2.0	0.29	0.01	0.46	0.28	0.85	12	1.9	0.35	1.7	1.0
HpL3-lab	6.3	3.1	0.42	0.01	0.59	0.31	0.93	16	1.9	0.34	2.3	1.5
HpL4-lab	4.7	2.1	0.40	0.01	0.78	0.32	0.76	13	3.0	0.46	1.6	1.3
HpL5-doc	5.3	2.0	0.17	0.01	0.54	0.36	0.97	7.7	3.0	0.47	1.7	1.1
HpL6-doc	5.2	3.3	0.20	0.01	0.41	0.32	1.5	17	2.1	0.36	2.2	1.8
CL1-lab	3.8	<LOQ	<LOQ	0.45	0.07	1.2	0.18	13	2.1	0.35	1.7	1.6
CL2-lab	3.9	2.2	0.34	0.01	0.69	0.23	0.42	13	2.4	0.41	1.8	0.92
CL3-lab	4.2	2.5	0.43	0.02	0.75	0.26	0.56	9.6	2.4	0.38	1.9	1.2
CL4-off	4.6	3.1	0.33	0.01	0.39	0.22	0.51	7.9	2.2	0.38	2.0	1.5
CL5-out	3.2	3.1	0.44	<LOQ	0.47	0.20	0.54	4.0	2.0	0.27	1.9	1.7
ParL1-lab	4.9	2.3	0.25	0.01	0.33	0.19	0.59	5.0	1.9	0.30	1.9	1.1
ParL2-lab	5.4	2.5	0.26	0.01	0.30	0.19	0.50	5.8	1.7	0.28	2.0	1.2
ParL3-corr	5.9	1.5	0.16	0.01	0.29	0.15	0.37	5.2	1.9	0.31	1.7	0.61
ParL4-lab	5.7	2.6	0.27	0.01	0.32	0.18	0.46	5.7	1.8	0.29	2.0	1.2

**Table 7:** Scores and variance % of the five components that were obtained by the PCA that was performed on the concentration data that were yielded at each work place.

	PC1	PC2	PC3	PC4	PC5
Variance%	47.4	20.9	14.5	8.5	4.5
PL1−lab	−2.49	−0.09	1.10	−0.82	−1.55
PL2−out	−2.22	−0.29	−0.39	0.65	0.12
PL3−tech	−2.32	0.02	1.45	−1.40	−2.13
PL4−tech	−2.01	−0.03	1.00	−1.33	−2.13
PL5−doc	−2.31	−0.04	1.25	−1.22	−1.83
PL6−solv	−2.20	−0.32	−0.49	0.60	0.22
GA1−nurs	−2.19	−0.33	−0.44	0.59	0.18
GA2−nurs	−2.33	−0.24	−0.32	0.70	0.13
GA3−doc	−1.39	−0.59	−0.01	0.28	0.54
GA4−lab	−2.37	−0.48	−0.34	0.64	0.27
GA5−out	−1.77	−0.66	−0.24	0.42	0.53
CS1−desk	−0.37	−0.32	0.56	−0.07	0.34
CS2−glue	−0.34	−0.26	0.82	−0.05	0.30
CS3−photo	−1.00	−0.11	0.29	0.34	0.34
BS1−hair	1.08	3.20	1.62	1.04	−0.19
BS2−nail	2.92	2.81	1.99	0.43	0.49
BS3−sol	2.49	3.45	1.75	0.85	−0.09
BS4−rec	1.60	3.00	1.16	0.84	−0.28
BS5−bea	4.42	4.60	2.88	0.80	0.46
BS6−out	0.13	0.50	0.24	0.25	0.18
PH1−out	−1.74	−0.19	−0.33	0.74	0.31
PH2−ware	−0.48	−0.05	0.08	0.06	0.13
PH3−store	−0.46	0.19	−0.08	0.12	0.14
PH4−dercon	−0.31	0.40	0.14	0.30	0.32
PH5−phar	−0.29	0.07	−0.05	0.05	0.32
PH6−der	−0.89	0.38	−0.04	0.33	−0.03
HL1−lab	8.91	−7.13	2.20	2.94	−1.04
HL2−tech	0.03	−1.74	1.32	−1.17	0.93
HL3−tech	1.21	−1.73	2.88	−3.43	0.18
HL4−sec	−0.68	−0.96	0.47	−0.58	0.83
HL5−tech	1.26	−1.92	2.33	−2.71	2.24
HL6−out	−2.12	−0.73	−0.22	0.46	0.64
HRL1−tech	1.15	0.02	−1.32	−0.27	0.20
HRL2−lab	−0.41	−0.47	−0.67	−0.29	0.89
HRL3−out	0.98	−0.15	−1.17	0.19	0.16
HRL4−bio	5.69	0.41	−2.97	−1.04	−0.43
HRL5−lab	8.23	1.38	−4.08	−2.41	−0.52
HpL1−out	−0.83	−0.07	−1.13	0.44	−0.04
HpL2−tech	−0.72	−0.16	−0.59	0.14	−0.06
HpL3−lab	−0.10	0.09	−0.81	−0.03	−0.40
HpL4−lab	−0.14	−0.34	−0.60	−0.23	0.20
HpL5−doc	−0.33	−0.08	−0.67	0.01	0.35
HpL6−doc	−0.15	0.36	−0.88	−0.16	−0.44
CL1−lab	−0.22	−0.98	0.18	1.41	−0.87
CL2−lab	−0.50	−0.29	−0.62	−0.12	0.08
CL3−lab	−0.25	−0.26	−0.83	0.00	0.11
CL4−off	−0.34	0.02	−1.19	0.06	−0.13
CL5−out	−0.36	−0.08	−1.42	0.17	−0.06
ParL1−lab	−0.78	0.05	−0.99	0.36	0.03
ParL2−lab	−0.78	0.08	−1.05	0.35	−0.09
ParL3−corr	−1.20	−0.05	−0.67	0.41	0.23
ParL4−lab	−0.72	0.10	−1.08	0.33	−0.08

**Table 8:** Loadings of the five components that were obtained by the PCA that were performed on the concentration data that were yielded at each workplace.

	PC1	PC2	PC3	PC4	PC5
Acetaldehyde	0.17	0.48	0.34	0.21	0.00
Butyraldehyde	0.29	0.10	−0.46	−0.26	−0.18
Cyclohexanone	0.32	−0.31	−0.17	0.15	−0.23
Crotonaldehyde	0.22	−0.40	0.17	0.45	−0.30
Decanal	0.29	−0.18	0.32	−0.24	0.50
Heptanal	0.37	−0.17	0.14	0.22	−0.14
Hexanal	0.27	0.41	0.27	0.17	0.08
Formaldehyde	−0.02	0.02	0.50	−0.58	−0.63
Nonanal	0.37	0.03	−0.14	−0.34	0.23
Octanal	0.32	−0.29	0.21	−0.17	0.24
Propionaldehyde	0.29	0.41	−0.05	0.17	−0.11
Valeraldehyde	0.35	0.15	−0.32	−0.14	−0.18

## Data Availability

All data are available upon request to the corresponding author.

## Supplementary Materials

The following supporting information can be downloaded at: https://www.mdpi.com/article/10.3390/ijerph191912052/s1, S1. LC-UV/DAD chromatogram of blank cartridge; S2. LC-MS/MS chromatogram of aldehydes-DNPH standard solution; S3. LC-UV/DAD chromatogram of aldehydes-DNPH standard solution; S4. LC-MS/MS and LC-UV/DAD chromatograms of PL5-doc; S5. LC-MS/MS and LC-UV/DAD chromatograms of BS1-hair; S6. LC-MS/MS and LC-UV/DAD chromatograms of ParL4-lab; S7. Aldehyde concentrations in blank samples.

## References

[1] Ho S.S.H., Cheng Y., Bai Y., Ho K.F., Dai W.T., Cao J.J., Lee S.C., Huang Y., Ip H.S.S., Deng W.J. (2016). Risk Assessment of Indoor Formaldehyde and Other Carbonyls in Campus Environments in Northwestern China. Aerosol Air. Qual. Res..

[2] De Carvalho A.B., Kato M., Rezende M.M., de Pereira P.A., de Andrade J.B. (2008). Determination of carbonyl compounds in the atmosphere of charcoal plants by HPLC and UV detection. J. Sep. Sci..

[3] Lewtas J. (2007). Air pollution combustion emissions: Characterization of causative agents and mechanisms associated with cancer, reproductive, and cardiovascular effects. Mutat. Res..

[4] Atkinson R., Arey J. (2003). Atmospheric degradation of volatile organic compounds. Chem. Rev..

[5] WHO (World Health Organization) (2010). WHO Guidelines for Indoor Air Quality. Selected Pollutants.

[6] Sousa F.W., Caracas I.B., Nascimento R.F., Cavalcante R.M. (2011). Exposure and cancer risk assessment for formaldehyde and acetaldehyde in the hospitals, Fortaleza-Brazil. Build Environ..

[7] IARC (International Agency for Research on Cancer) (1999). Monographs on the Evaluation of Carcinogenic Risks to Humans. Re-Evaluation of some Organic Chemicals, Hydrazine and Hydrogen Peroxide.

[8] IARC (International Agency for Research on Cancer) (2006). Monographs on the Evaluation of Carcinogenic risks to Humans. Formaldehyde, 2-Butoxyethanol and 1-Tert-Butoxypropan-2-ol.

[9] Protano C., Buomprisco G., Cammalleri V., Pocino R.N., Marotta D., Simonazzi S., Cardoni F., Petyx M., Iavicoli S., Vitali M. (2022). The Carcinogenic Effects of Formaldehyde Occupational Exposure: A Systematic Review. Cancers.

[10] Hadei M., Hopke P.K., Shahsavani A., Moradi M., Yarahmadi M., Emam B., Rastkari N. (2018). Indoor concentrations of VOCs in beauty salons; association with cosmetic practices and health risk assessment. J. Occup. Med. Toxicol..

[11] Jafari M.J., Khajevandi A.A., Mousavi Najarkola S.A., Yekaninejad M.S., Pourhoseingholi M.A., Omidi L., Kalantary S. (2015). Association of sick building syndrome with indoor air parameters. Tanaffos.

[12] EPA (United States Environmental Protection Agency) (2015). Questions about Your Community: Indoor Air.

[13] Cammalleri V., Pocino R.N., Marotta D., Protano C., Sinibaldi F., Simonazzi S., Petyx M., Iavicoli S., Vitali M. (2022). Occupational scenarios and exposure assessment to formaldehyde: A systematic review. Indoor Air.

[14] Scarselli A., Corfiati M., Di Marzio D., Iavicoli S. (2017). National Estimates of Exposure to Formaldehyde in Italian Workplaces. Ann. Work Expo. Health.

[15] Jurvelin J., Vartiainen M., Jantunen M., Pasanen P. (2001). Personal Exposure Levels and Microenvironmental Concentrations of Formaldehyde and Acetaldehyde in the Helsinki Metropolitan Area, Finland. J. Air Waste Manag. Assoc..

[16] Maroni M., Seifert B., Lindvall T. (1995). Indoor Air Quality a Comprehensive Reference Book.

[17] Calisti R., Chiaverini A.F., Mei R. (2020). Aldehydes others than formaldehyde: Exposure patterns and their significance in a set of Italian indoor workplaces, 2011–2020. J. Occup. Environ. Hyg..

[18] TOXNET (2020). Crotonaldehyde.

[19] Zhang S., Chen H., Wang A., Liu Y., Hou H., Hu Q. (2017). Assessment of genotoxicity of four volatile pollutants from cigarette smoking based on the in vitro yH2AX assay using high content screening. Environ. Toxicol. Pharmacol..

[20] Zhang S., Chen H., Wang A., Liu Y., Hou H., Hu Q. (2018). Combined effect of co-exposure to formaldehyde and acrolein mixtures on cytotoxicity and genotoxicity in vitro. Environ. Sci. Pollut. Res. Int..

[21] Xie M.Z., Shoulkamy M.I., Salem A.M., Oba S., Goda M., Nakano T., Ide H. (2016). Aldehydes with high and low toxicities inactivate cells by damaging distinct cellular targets. Mutat. Res..

[22] Chi Y., Feng Y., Wen S., Lü H., Yu Z., Zhang W., Sheng G., Fu J. (2007). Determination of carbonyl compounds in the atmosphere by DNPH derivatization and LC-ESI-MS/MS detection. Talanta.

[23] Zeng Y., Wen S., Chen Y., Wang X., Lü H., Bi X., Sheng G., Fu J. (2005). Ambient levels of carbonyl compounds and their sources in Guangzhou, China. Atmos. Environ..

[24] Wang H.W., Tong X.Y., Yan L.Q., Sheng J.Y., Liu S.M. (2005). Determination of Volatile Carbonyl Compounds in Cigarette Smoke by LC-DAD. Chromatographia.

[25] Zhang T., Jiang G., Guazzotti S., Elmashni D. (2010). Quantitative Analysis of Carbonyl-DNPH Derivatives by UHPLC/UV.

[26] Lü H., Wen S., Feng Y., Wang X., Bi X., Sheng G., Fu J. (2006). Indoor and outdoor carbonyl compounds and BTEX in the hospitals of Guangzhou, China. Sci. Total Environ..

[27] Lü H., Cai Q.Y., Wen S., Chi Y., Guo S., Sheng G., Fu J., Katsoyiannis A. (2010). Carbonyl compounds and BTEX in the special rooms of hospital in Guangzhou, China. J. Hazard Mater..

[28] Evtyugina M., Vicente E.D., Vicente A.M., Nunes T., Lucarelli F., Calzolai G., Nava S., Blanco-Alegre C., Calvo A.I., Castro A. (2021). Air quality and particulate matter speciation in a beauty salon and surrounding outdoor environment: Exploratory study. Atmos. Pollut. Res..

[29] Perdelli F., Spagnolo A.M., Cristina M.L., Sartini M., Dallera M., Ottria G., Orlando P. (2006). Occupational exposure to formaldehyde in three pathology departments. Ann. Ig..

[30] De Ochs M., Grotz L.O., Factorine L.S., Rodrigues M.R., Pereira Netto A.D. (2011). Occupational exposure to formaldehyde in an institute of morphology in Brazil: A comparison of area and personal sampling. Environ. Sci. Pollut. Res. Int..

[31] Bellisario V., Mengozzi G., Grignani E., Bugiani M., Sapino A., Bussolati G., Bono R. (2016). Towards a formalin-free hospital. Levels of 15-F2t-isoprostane and malondialdehyde to monitor exposure to formaldehyde in nurses from operating theatres. Toxicol. Res..

[32] Higashikubo I., Miyauchi H., Yoshida S., Tanaka S., Matsuoka M., Arito H., Araki A., Shimizu H., Sakurai H. (2017). Assessment of workplace air concentrations of formaldehyde during and before working hours in medical facilities. Ind. Health.

[33] Lee E.G., Magrm R., Kusti M., Kashon M.L., Guffey S., Costas M.M., Boykin C.J., Harper M. (2017). Comparison between active (pumped) and passive (diffusive) sampling methods for formaldehyde in pathology and histology laboratories. J. Occup. Environ. Hyg..

[34] Saraga D., Pateraki S., Papadopoulos A., Vasilakos C., Maggos T. (2011). Studying the indoor air quality in three non-residential environments of different use: A museum, a printery industry and an office. Build Environ..

[35] Loh M.M., Houseman E.A., Gray G.M., Levy J.I., Spengler J.D., Bennett D.H. (2006). Measured Concentrations of VOCs in Several Non-Residential Microenvironments in the United States. Environ. Sci. Technol..

[36] Liang W., Zhao B., Liu J., Pei J. (2020). Can carbon dioxide be a good indicator for formaldehyde in residences?—Monte Carlo modeling for a whole year. Sci. Technol. Built. Environ..

[37] Shao Y., Wang Y., Zhao R., Chen J., Zhang F., Linhardt R.J., Zhong W. (2020). Biotechnology progress for removal of indoor gaseous formaldehyde. Appl. Microbiol. Biotechnol..

