# Supplementary material for: Determination of Carbonyl Compounds in Different Work Environments: Comparison between LC-UV/DAD and LC–MS/MS Detection Methods
Source: Int J Environ Res Public Health. 2022 Sep 23;19(19):12052. doi: 10.3390/ijerph191912052 (PMC9565147; doi:10.3390/ijerph191912052)
Supplement: Supplementary file 1 [file ijerph-19-12052-s001.zip › ijerph-1882102-supplementary.pdf]

Determination of Carbonyl Compounds in Different Work Environments: Comparison between LC-UV/DAD and LC–MS/MS Detection Methods

Federica Castellani [1,2], Arianna Antonucci [1], Ivano Pindinello [1], Carmela Protano [1], Matteo Vitali [1,*]

[1] Department of Public Health and Infectious Diseases, Sapienza University of Rome, P.le Aldo Mo-ro, 5, 00185 Rome, Italy

[2] Department of Ecological and Biological Sciences, Tuscia University, Largo dell'Università snc, 01100 Viterbo, Italy

*Correspondence: matteo.vitali@uniroma1.it; Tel.: +39-0649914560

S1. LC-UV/DAD chromatogram of blank cartridge

S2. LC-MS/MS chromatogram of aldehydes-DNPH standard solution

S3. LC-UV/DAD chromatogram of aldehydes-DNPH standard solution

S4. LC-MS/MS and LC-UV/DAD chromatograms of PL5-doc

S5. LC-MS/MS and LC-UV/DAD chromatograms of BS1-hair

S6. LC-MS/MS and LC-UV/DAD chromatograms of ParL4-lab

S7. Aldehyde concentrations in blank samples

S1. LC-UV/DAD chromatogram of blank cartridge

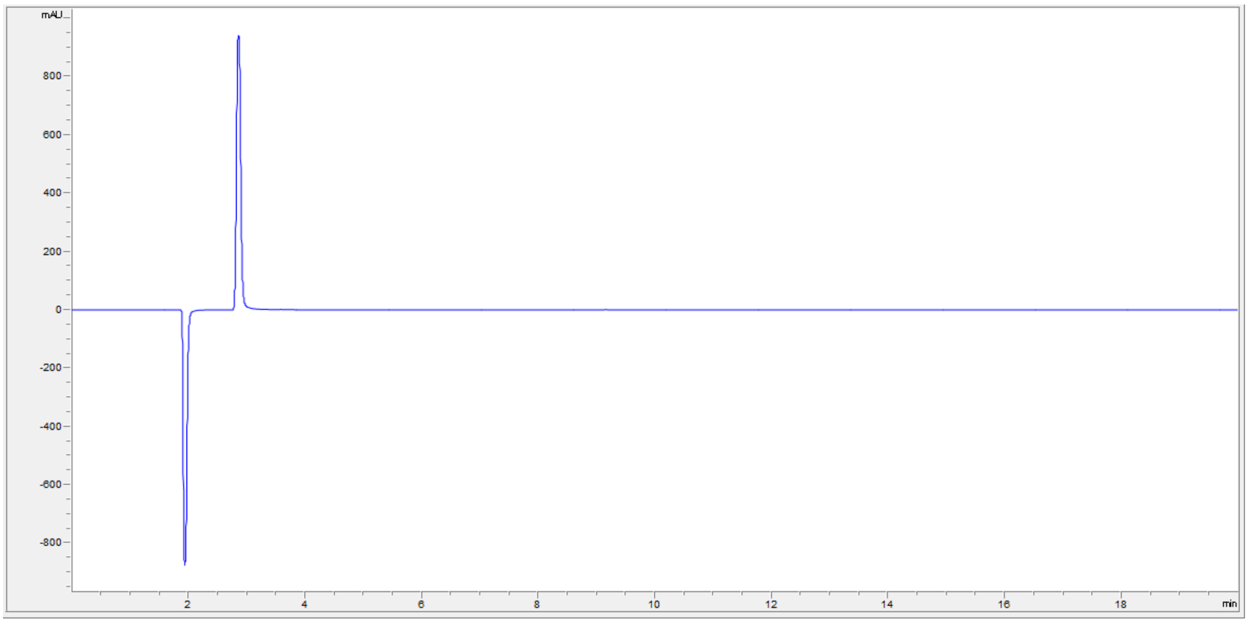

Figure S1. LC-UV/DAD chromatogram of blank cartridge. The peak at 3.85 min is the unreacted DNPH.

S2. LC-MS/MS chromatogram of aldehydes-DNPH standard solution

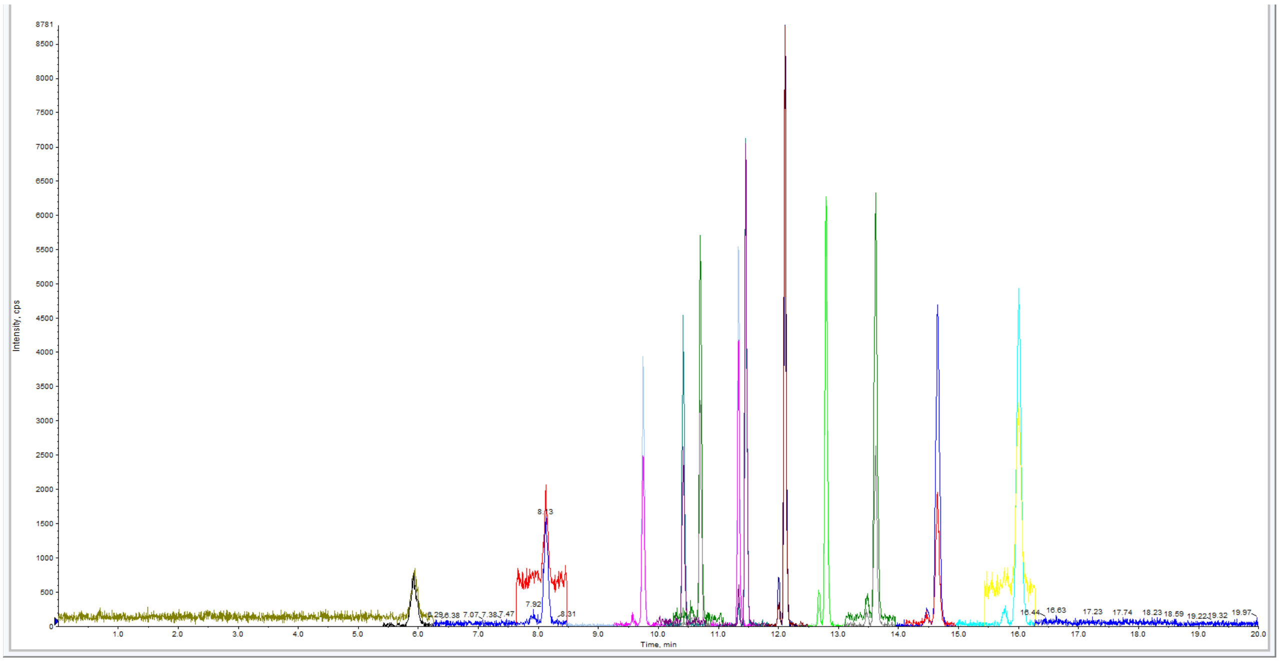

Figure S2. LC-MS/MS chromatogram obtained by injecting 5 µL of aldehydes-DNPH standard solution at 0.2 µg L^{-1}. Peaks identification: Fo-DNPH (5.83 min), Ace-DNPH (8.05 min), Pro-DNPH (9.68 min), Cro-DNPH (10.36 min), Bu-DNPH (10.65 min), Cy-DNPH (11.29 min), Val-DNPH (11.40 min), Hex-DNPH (12.06 min), Hep-DNPH (12.73 min), Oct-DNPH (13.53 min), Non-DNPH (14.53 min), Dec-DNPH (15.85 min).

S3. LC-UV/DAD chromatogram of aldehydes-DNPH standard solution

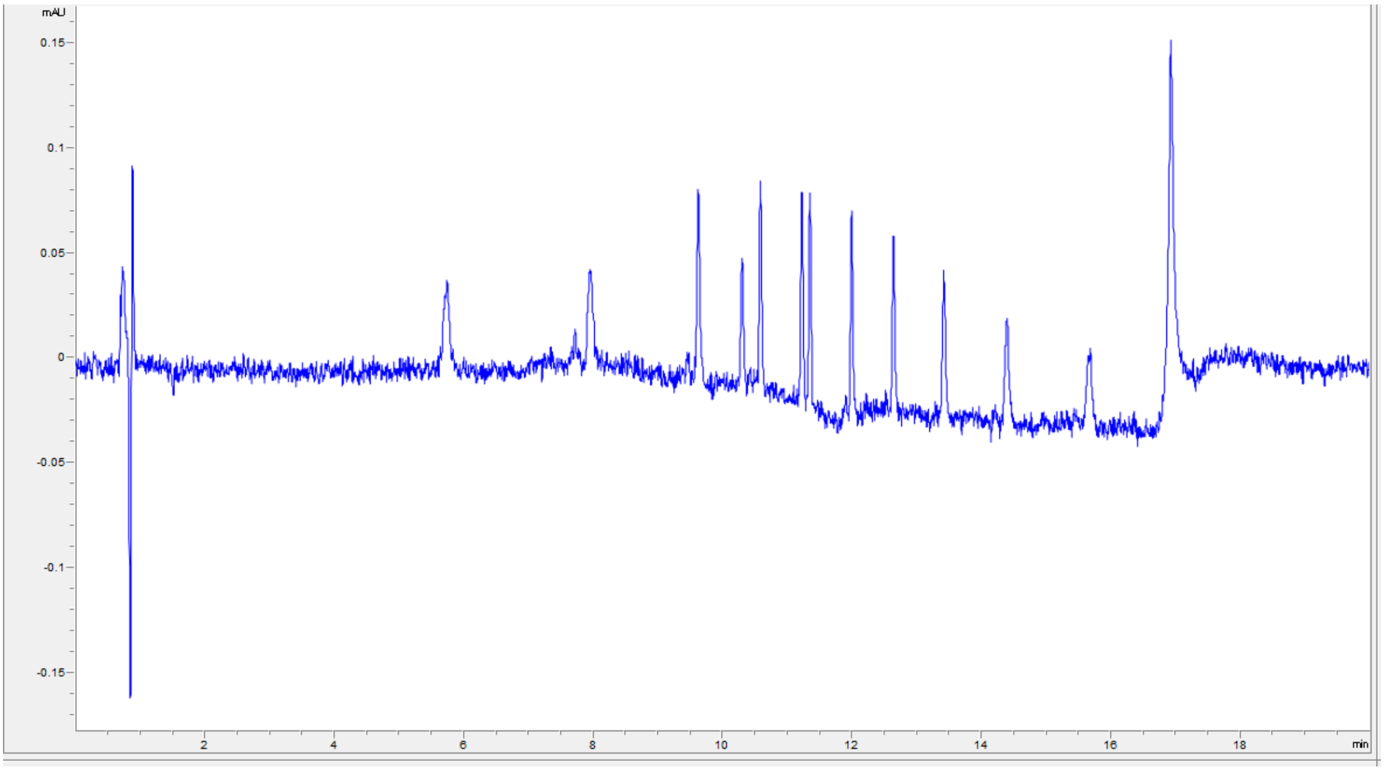

Figure S3. LC-UV/DAD chromatogram obtained by injecting 5 µL of aldehydes-DNPH standard solution at 25 µg L^{-1}. Peaks identification: Fo-DNPH (5.83 min), Ace-DNPH (8.05 min), Pro-DNPH (9.68 min), Cro-DNPH (10.36 min), Bu-DNPH (10.65 min), Cy-DNPH (11.29 min), Val-DNPH (11.40 min), Hex-DNPH (12.06 min), Hep-DNPH (12.73 min), Oct-DNPH (13.53 min), Non-DNPH (14.53 min), Dec-DNPH (15.85 min).

S4. LC-MS/MS and LC-UV/DAD chromatograms of PL5-doc

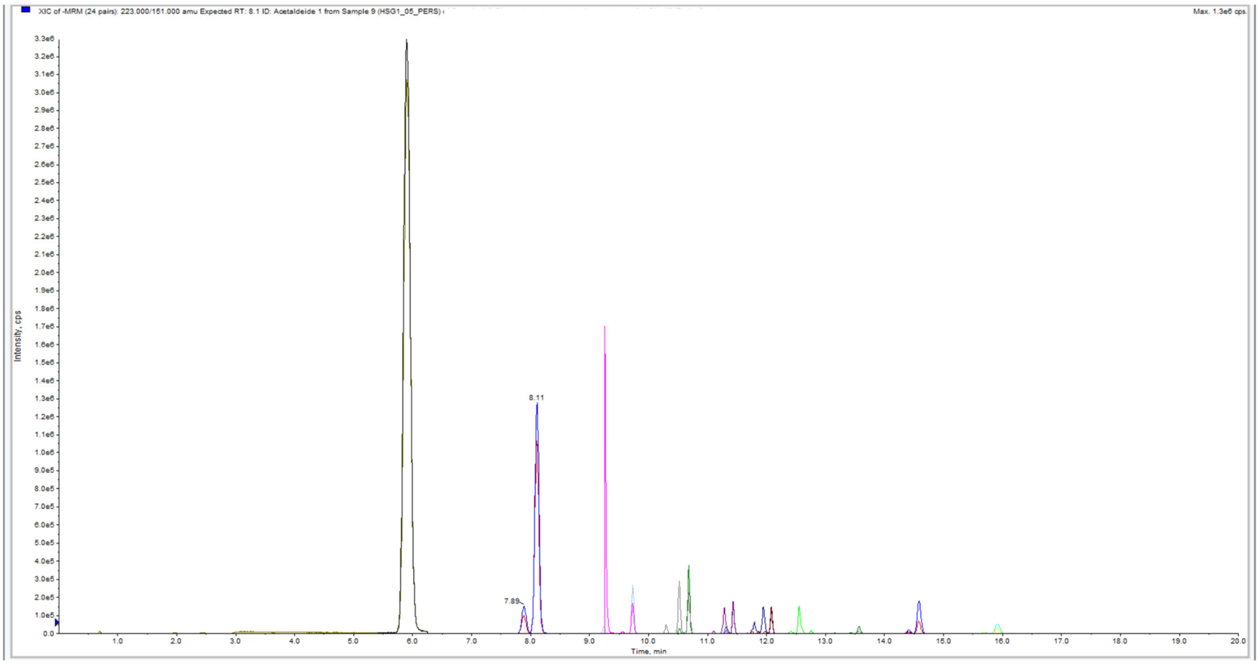

Figure S4-1. LC-MS/MS chromatogram obtained by injecting 5 µL of the extracted sample PL5-doc.

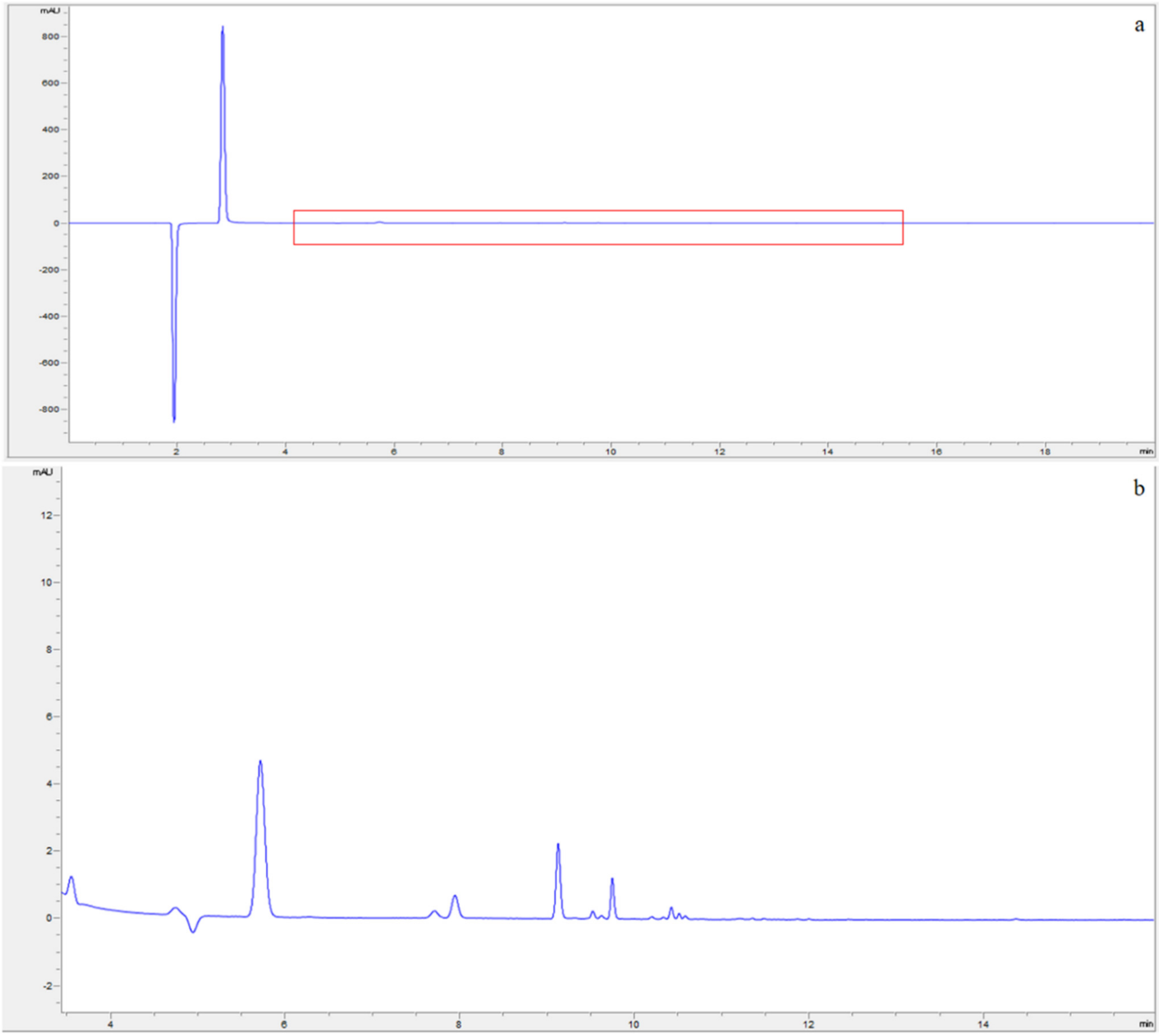

Figure S4-2. LC-UV/DAD chromatogram obtained by injecting 5 µL of the extracted sample PL5-doc. The Figure S4-2a represent the total chromatogram. The figure S4-2b is an enlargement of figure S4-2a.

S5. LC-MS/MS and LC-UV/DAD chromatograms of BS1-hair

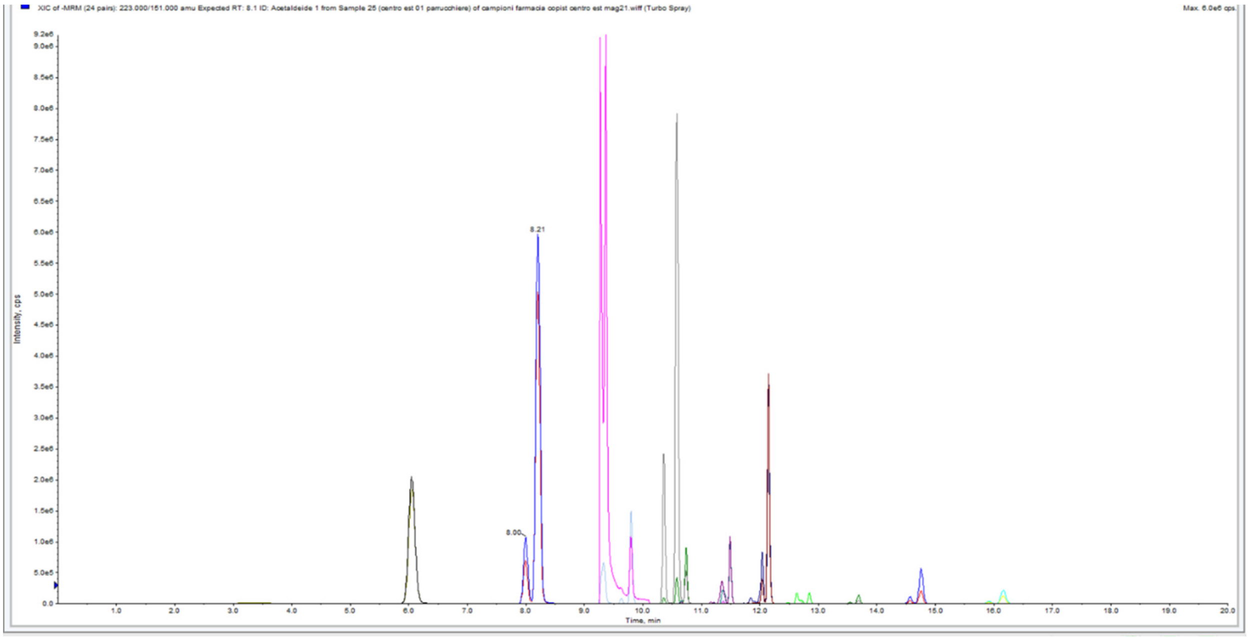

Figure S5-1. LC-MS/MS chromatogram obtained by injecting 5 µL of the extracted sample BS1-hair.

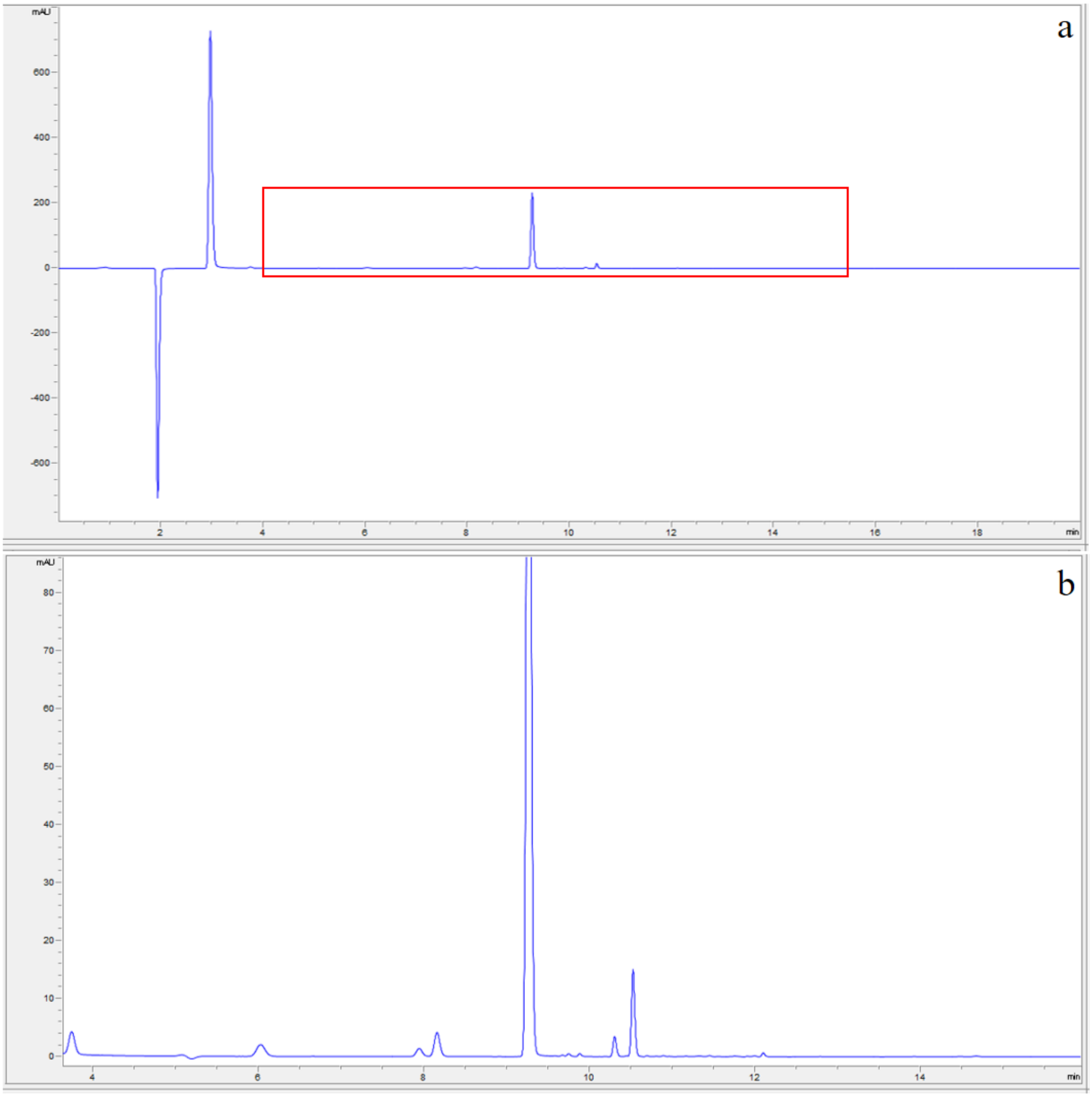

Figure S5-2. LC-UV/DAD chromatogram obtained by injecting 5 µL of the extracted sample BS1-hair. The Figure S5-2a represent the total chromatogram. The figure S5-2b is an enlargement of figure S5-2a.

S6. LC-MS/MS and LC-UV/DAD chromatograms of ParL4-lab

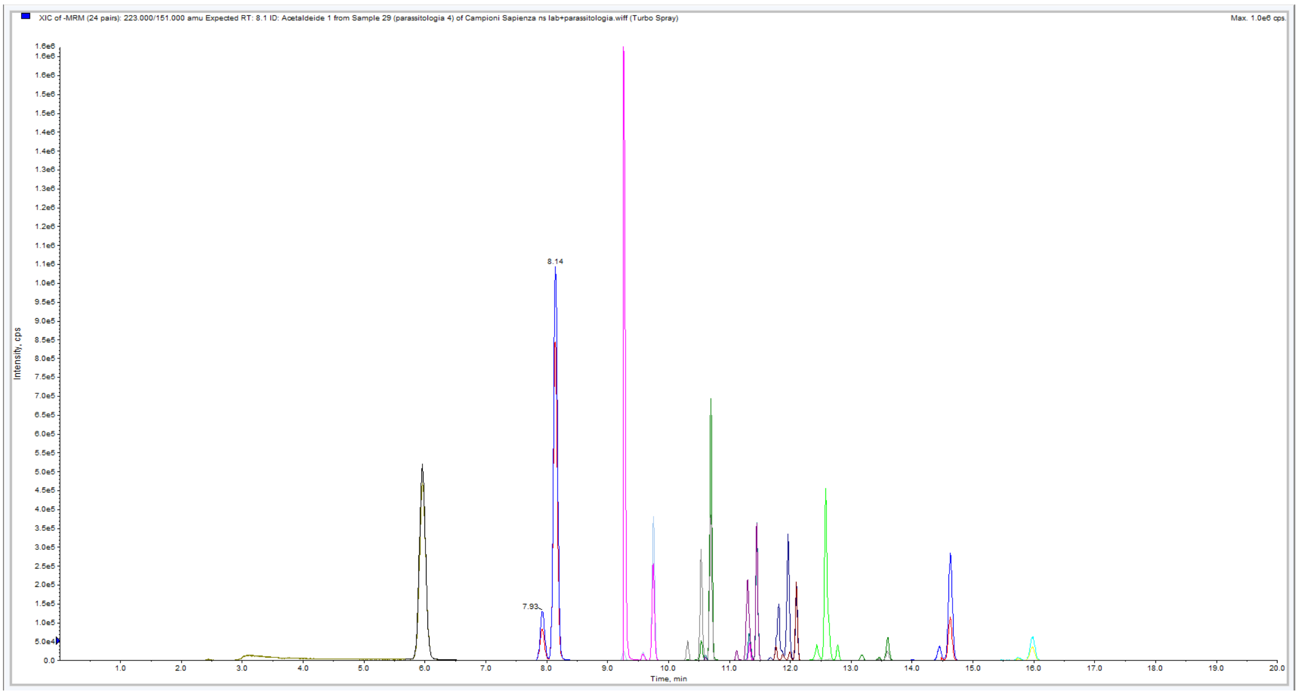

Figure S6-1. LC-MS/MS chromatogram obtained by injecting 5 µL of the extracted sample ParL4-lab.

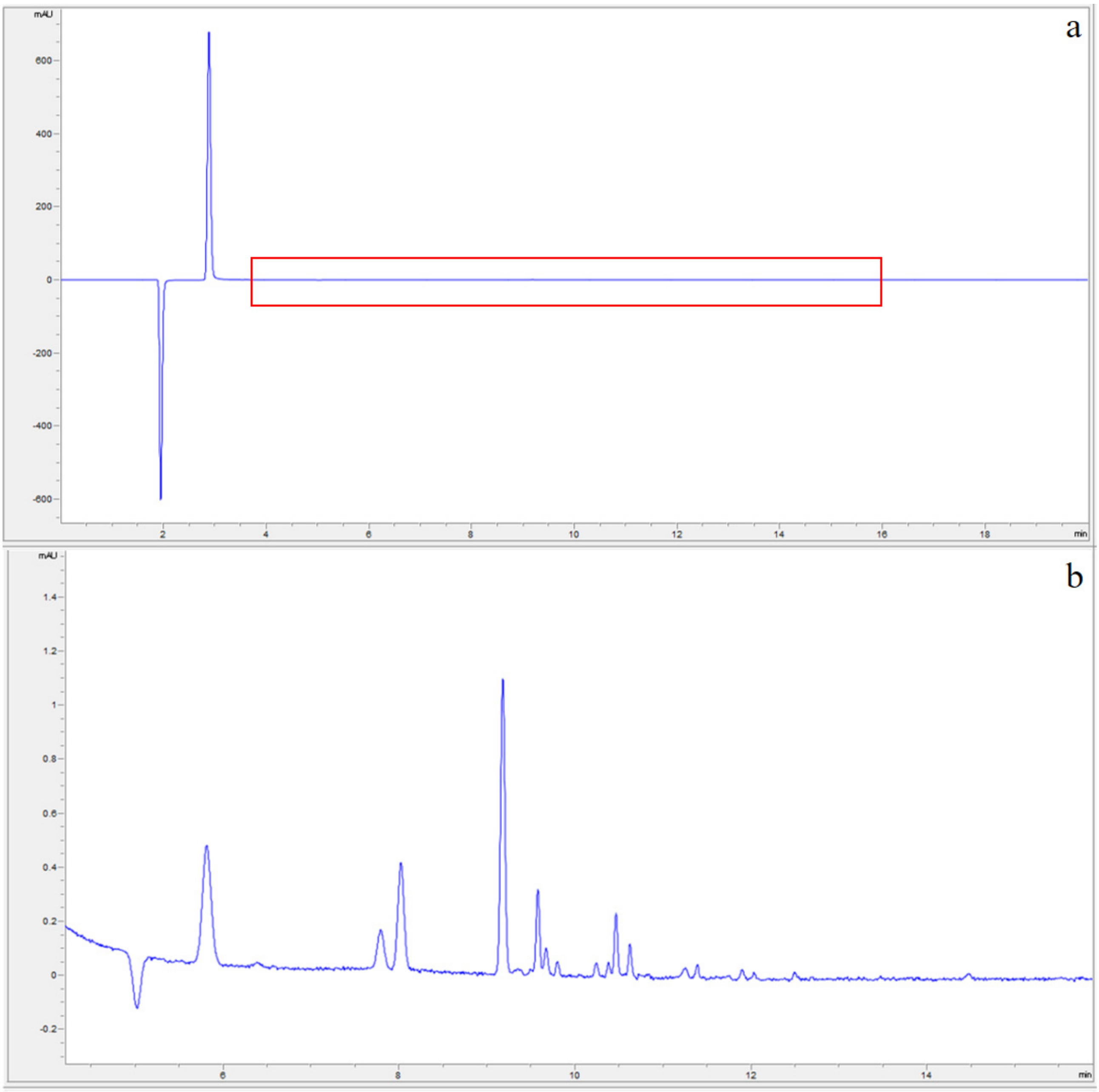

Figure S6-2. LC-UV/DAD chromatogram obtained by injecting 5 µL of the extracted sample ParL4-lab. The Figure S6-2a represent the total chromatogram. The figure S6-2b is an enlargement of figure S6-2a.

S7. Aldehyde concentrations in blank samples

Table S7. Average concentration (μg L^{-1}), standard deviation (SD) and relative standard deviation (RSD%) obtained from the analysis of blank cartridges (n = 5) using both LC-MS/MS and LC-UV/DAD detection methods.

	LC-MS/MS			LC-UV/DAD		
	**Average concentration**	**SD**	**RSD%**	**Average concentration**	**SD**	**RSD%**
**Fo-DNPH**	173.7	2.5	1	150.1	10.9	7
**Ace-DNPH**	191.4	4.9	3	149.5	4.9	3
**Pro-DNPH**	17.0	0.5	3	< LOQ		
**Cro-DNPH**	< LOQ			< LOQ		
**Bu-DNPH**	15.8	0.5	3	< LOQ		
**Cy-DNPH**	3.4	0.2	5	nd		
**Val-DNPH**	12.3	0.6	5	< LOQ		
**Hex-DNPH**	13.0	0.7	6	< LOQ		
**Hep-DNPH**	1.7	0.3	18	nd		
**Oct-DNPH**	3.5	0.2	5	nd		
**Non-DNPH**	24.1	1.4	6	< LOQ		
**Dec-DNPH**	6.5	0.3	5	nd		
